# The Impact of Vaccination as a Strategy to Combat Bacterial Antimicrobial Resistance

**DOI:** 10.7759/cureus.65840

**Published:** 2024-07-31

**Authors:** Esteban Zavaleta-Monestel, Samuel Hasselmyr Hasselmyr, Jonathan García-Montero, Sebastián Arguedas-Chacón, Carolina Rojas-Chinchilla, José Pablo Díaz-Madriz

**Affiliations:** 1 Pharmacy, Hospital Clinica Biblica, San Jose, CRI; 2 Faculty of Pharmacy, Universidad de Iberoamerica Costa Rica, San Jose, CRI; 3 Pharmacy and Clinical Research, Hospital Clinica Biblica, San Jose, CRI

**Keywords:** antimicrobials, public health interventions, antimicrobial resistance, antimicrobial stewardship program, vaccination

## Abstract

Antimicrobial resistance (AMR) poses a significant threat to global health, impairing the efficacy of treatments against various infections. The World Health Organization highlights the impact of AMR on healthcare outcomes, including increased morbidity, mortality, and costs. Vaccination is a pivotal strategy to counter AMR, promoting immune defenses against infections and subsequently reducing the need for antimicrobials. This article assesses the role of vaccination in managing AMR, particularly within the scope of antimicrobial stewardship programs (ASPs), by reviewing the effectiveness of existing vaccination strategies and their integration into the community. A comprehensive literature review was concluded using databases such as Google Scholar, Scielo, and PubMed, analyzing studies from 2005 to 2024. A total of 13 studies were included after screening 132 articles for relevance and eligibility. The studies highlight the substantial role of vaccines in reducing the reliance on antibiotics, especially for vulnerable populations, such as the elderly, children, and those with chronic conditions. For instance, the introduction of conjugate pneumococcal vaccines significantly decreased rates of drug-resistant Streptococcus pneumoniae infections. The review also discusses the indirect benefits of widespread vaccination, including herd immunity and decreased transmission of resistant strains. Vaccination is a critical element in the fight against AMR. Well-coordinated ASPs, by facilitating comprehensive vaccination programs, can significantly mitigate the rise of resistant infections, optimize antimicrobial usage, and improve patient outcomes in healthcare settings. The strategies outlined reflect global health objectives and emphasize the need for sustained efforts to enhance vaccine coverage and acceptance.

## Introduction and background

Antimicrobial resistance (AMR) is a global public health issue that threatens the effectiveness of available treatments for bacterial, viral, parasitic, and fungal infections [1]. AMR occurs when microorganisms change in a way that the drugs used to treat them become ineffective or less effective [2]. According to the World Health Organization (WHO), AMR contributes to increased morbidity, mortality, prolonged hospital stays, and elevated healthcare costs [3].

Vaccination represents a critical strategy in combating AMR, involving the administration of substances containing antigens that stimulate specific immune responses against infectious agents [4]. Successful vaccination programs have demonstrated substantial reductions in antimicrobial use by preventing primary and secondary infections, interrupting transmission chains, and mitigating the emergence of resistant strains [5]. For instance, the introduction of Haemophilus influenzae type b (Hib) conjugate vaccines in the late 1980s marked a turning point in pediatric infectious disease management. Hib vaccines not only drastically reduced invasive Hib disease but also led to a significant decline in antibiotic use and the prevalence of antibiotic-resistant strains globally. Similarly, pneumococcal conjugate vaccines have shown over 90% efficacy in preventing invasive pneumococcal disease (IPD) in children, reducing antibiotic-resistant pneumococcal strains [6].

Moreover, influenza vaccines not only prevent influenza infections but also significantly reduce the incidence of secondary bacterial infections such as pneumonia and otitis media. A notable study conducted in Canada highlighted the impact of universal influenza vaccination on antibiotic use. Following the introduction of universal influenza vaccination in Ontario, the rate of influenza-associated antibiotic prescriptions decreased by approximately 64% compared to other provinces where vaccination was not universally implemented [6]. In addition, vaccination can have an indirect or herd effect by protecting unvaccinated people by interrupting transmission chains [7].

However, vaccination efforts encounter challenges, particularly in hospital settings where patients, due to factors such as advanced age, chronic illnesses, immunosuppression, or recent surgeries, are at heightened risk of infections and associated complications [8]. These challenges include inadequate awareness of vaccination benefits, logistical hurdles in vaccine access, negative perceptions towards vaccination, and periodic shortages of vaccine supplies [9].

This article aims to analyze the impact of vaccination as a strategy against AMR. To achieve this goal, it will review compelling evidence from successful vaccination initiatives that have influenced AMR and infection prevention worldwide. Furthermore, it will identify vaccination strategies that can be integrated into antimicrobial stewardship programs (ASPs).

## Review

Methods

A literature review was conducted to observe the impact of vaccination as a strategy against AMR. The following keywords were used in the search: "Vaccination", "Antimicrobial resistance", and "Antimicrobial Stewardship Programs " in the databases Google Scholar, Clinical Key, and PubMed, between 2005 and 2024.

Study Selection

**Figure 1:**
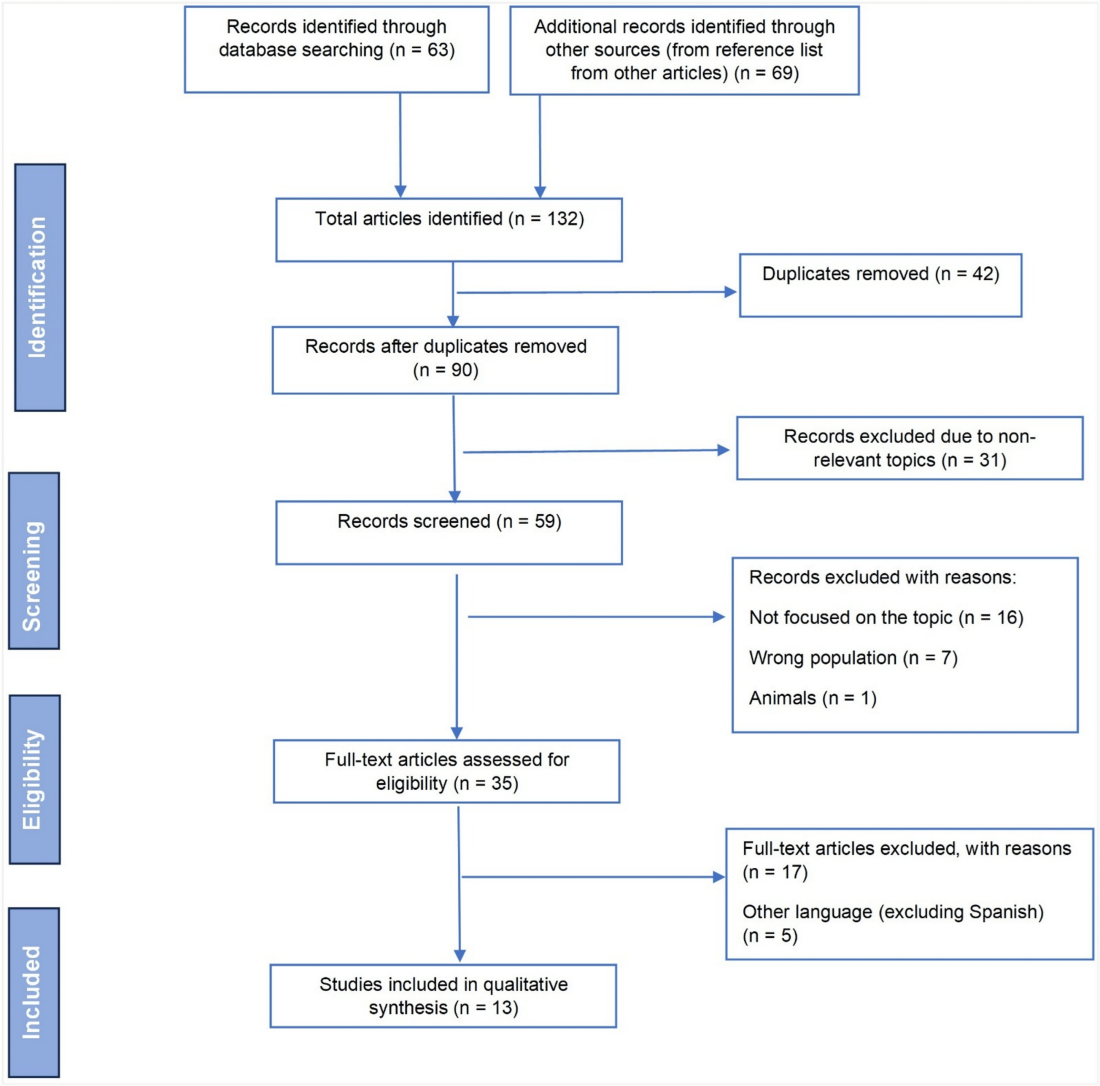
PRISMA flow chart for study selection. PRISMA: Preferred Reporting Items for Systematic Reviews and Meta-Analyses

A total of 63 records were identified by searching several databases. Additionally, 69 records were identified through other sources (reference lists from other articles), resulting in a total of 132 articles. After the removal of duplicates and records excluded from non-relevant topics, 59 records were considered for screening. Then, excluding records due to the wrong population, and not focused on the topic, 35 full texts were assessed for eligibility. Thereafter, certain full texts were excluded with reason, and due to languages (other than English and Spanish), ending up with 13 articles that were included in the review (Figure 1).

Results

The characteristics of the included studies are presented in Table 1. These 13 articles collectively emphasize the importance of integrating vaccination strategies within ASPs to combat antibiotic resistance and improve patient outcomes. They highlight the practicality and effectiveness of vaccination over restrictive antibiotic policies, showing a reduction in their use in both general and specific patient groups, including the elderly, children, and those with chronic conditions such as chronic obstructive pulmonary disease. Additionally, the discussion extends to the potential of vaccines to reduce the overall need for antibiotics in the community and healthcare settings, thereby reducing antibiotic utilization that leads to AMR. They also touch on future strategies and the global initiative, as seen in the WHO's 2030 agenda, to improve vaccine-related interventions as a central component of antimicrobial stewardship efforts.

**Table 1:** Characteristics of the included studies of vaccination and its effects on ASPs and AMR. ASP: Antimicrobial Stewardship Program, PVC7/PVC13: Pneumococcal Conjugate Vaccine (7-valent/13-valent), IPD: Invasive Pneumococcal Disease, AMR: Antimicrobial Resistance, US: United States, WHO: World Health Organization, COPD: Chronic Obstructive Pulmonary Disease

Author Name	Year	Journal	Study Design	Study Setting	Content Summary
O’Brien et al. [10]	2013	Current Opinion in Infectious Diseases	Review	UK	Vaccines help to reduce the prevalence of diseases and the reliance on antibiotics, highlighting their importance in ASP strategies. Specific examples include pneumococcal conjugate vaccines (PCVs), which have significantly reduced antibiotic-resistant pneumococcal infections. These vaccines directly impact bacterial pathogens, demonstrating their role in lowering antibiotic use and combating resistance. Additionally, viral vaccines such as influenza vaccines indirectly contribute by preventing secondary bacterial infections, thereby supporting ASP efforts.
Wilby et al. [11]	2012	Elsevier	Review	Canada	A comprehensive search identified seven relevant articles, including three randomized controlled trials and four epidemiological studies, focusing on pneumococcal and influenza immunization programs, assessing vaccine effectiveness. Studies consistently demonstrated reduced antibiotic use following the initiation or increased uptake of vaccines. Specifically, pneumococcal conjugate vaccines (PCVs) and influenza vaccines were prominently featured. Randomized controlled trials reported reductions in antibiotic utilization ranging from 5% to 10%, while epidemiological studies indicated relative reductions of up to 64%. These findings underscore the potential of immunization programs to significantly decrease antibiotic use.
von Gottberg et al. [12]	2014	N Engl J Med	Descriptive study	South Africa	Impact of pneumococcal conjugate vaccines (PCV7 and PCV13) on invasive pneumococcal disease (IPD) rates showed significant reductions in IPD were observed among children under 2 years and adults aged 25–44 years, highlighting the role of vaccination in reducing disease incidence and antimicrobial resistance.
Darazam et al. [13]	2019	Arch Clin Infect Dis	Review	Iran	The introduction of universal influenza vaccination and postnatal vaccination of mothers has significantly reduced antibiotic prescriptions, providing conclusive evidence for annual influenza vaccination programs. Influenza virus can lead to mild acute respiratory illnesses or severe systemic and respiratory diseases, contributing to a considerable number of respiratory syndromes and complications. Influenza vaccination not only prevents millions of illnesses and flu-related visits annually but also reduces the incidence of bacterial complications post-influenza. The vaccination program indirectly diminishes antibiotic use by decreasing the incidence of flu-related bacterial infections, thereby reducing bacterial exposure to antibiotics and selective pressure for resistance.
AGPCIH [14]	2019	NHMRC	Guideline	Australia	Antimicrobial Stewardship Programs (ASPs) play a critical role in ensuring appropriate antimicrobial use and combating resistance. Vaccination and preventive measures, as recommended by the NHMRC guidelines, are integral to reducing infections and supporting ASP goals. Specific vaccines such as pneumococcal conjugate vaccines (PCVs) and influenza vaccines are highlighted in the guidelines for their efficacy in preventing bacterial and viral infections. PCVs have been effective in reducing pneumococcal infections, thereby lowering the need for antibiotics and mitigating resistance pressures. Influenza vaccination programs not only prevent influenza-related complications but also reduce the incidence of secondary bacterial infections, contributing further to antimicrobial stewardship efforts.
Wuethrich et al. [15]	2021	Taylor & Francis Group	Review	Germany	Vaccines reduce the use of antibiotics and the selection of resistant strains in the intestinal microbiota, highlighting their essential role in the fight against antimicrobial resistance.
SAAGAR [16]	2020	SAHealth	Expert opinion	Australia	An ASP intervention led to 172 inpatients being vaccinated for influenza through record screening for eligibility, with plans to expand this at discharge. Additionally, activities like Antibiotic Awareness Week were coordinated to promote safe antibiotic use. These initiatives highlight the integration of vaccination into ASPs, reducing antibiotic misuse and combating antimicrobial resistance due to that the influenza vaccines help prevent influenza infections and reduce the risk of secondary bacterial infections.
Smith et al. [17]	2019	Open Forum Infectious Diseases	Retrospective study	USA	Influenza vaccination has improved antibiotic prescribing by preventing 10.6% of acute respiratory infections and reducing antibiotic prescriptions by 7.3% in these cases. Influenza vaccines not only prevent influenza infections and disease, but also decrease the likelihood of secondary bacterial infections. Expanding the coverage of this vaccination could be crucial in reducing antimicrobial resistance at a national level.
Andrejko et al. [18]	2021	Lancet Microbe	Meta-analysis	83 countries	Data extracted from 559 studies covering 312,783 pediatric pneumococcal isolates from 2000 to 2020 revealed significant reductions in resistance post-PCV implementation. On average across all regions, there were substantial absolute reductions in the proportions of pneumococci showing non-susceptibility to penicillin (11.5%), sulfamethoxazole-trimethoprim (9.7%), and third-generation cephalosporins (7.5%) over the 10 years following any PCV product introduction. This underscores the importance of PCVs in combating AMR and the need for continued investment in vaccine coverage.
Buchy et al. [19]	2020	Int J Infect Dis	Review	Singapore	Integrating vaccination strategies is crucial for optimizing antibiotic use and combating antimicrobial resistance (AMR). For example, pneumococcal conjugate vaccines (PCVs) have reduced antibiotic-resistant infections by targeting specific serotypes, such as PCV7's 76% decline in invasive pneumococcal diseases among children under 5 years. Hib vaccines similarly decreased antibiotic-resistant strains, lowering β-lactamase-positive Hib cases after vaccination. Moreover, universal influenza vaccination in Ontario led to a 64% decrease in respiratory antibiotic prescriptions, illustrating how vaccination reduces antibiotic use. While vaccines mitigate AMR, ongoing surveillance and integrated strategies are essential to monitor effectiveness and combat resistance comprehensively.
Brink [20]	2016	Clinical Pulmonary Medicine	Review	South Africa	Vaccination is key in ASP, reducing antibiotic use and AMR. PCV7 in the US cut antibiotic prescriptions for otitis media by 41.9%, and PCV13 could reduce cases by 16.3 million. In South Africa, PCVs decreased penicillin-resistant infections by 82%, and ceftriaxone-resistant infections by 85%. Influenza vaccination in Ontario led to a 64% drop in antibiotic prescriptions.
WHO/Vekemans J et al. [21]	2021	Clin Infect Dis	Retrospective study	Switzerland	The WHO's Immunization Agenda for 2030 underscores vaccines' role in combatting antimicrobial resistance (AMR) by preventing infections and reducing antibiotic use. Pneumococcal conjugate vaccine 9 (PCV9) demonstrated a 67% decrease in penicillin-resistant invasive pneumococcal diseases in South Africa, highlighting its impact. Vaccines like PCV and rotavirus vaccines annually prevent millions of antibiotic-treated illnesses in children in low- and middle-income countries. Influenza vaccines reduce antibiotic use days in adults by 28.1%. Strategic efforts by organizations like WHO and Wellcome Trust emphasize prioritizing vaccines to tackle antibiotic-resistant pathogens, necessitating comprehensive evidence collection and transparent decision-making to address AMR effectively.
Feldman et al. [22]	2018	F1000Res	Meta-analysis	South Africa	ASPs are essential for managing lung diseases and reducing antibiotic resistance. Vaccination, particularly against Haemophilus influenzae in patients with COPD, has proven to reduce antibiotic use by 80%.
Kwong et al. [23]	2009	Clin Infect Dis	Review	Canada	Ontario's introduction of universal influenza immunization in 2000 led to a substantial 64% reduction in antibiotic prescriptions for respiratory illnesses compared to targeted vaccination strategies in other Canadian provinces. This underscores the effectiveness of universal vaccination in curbing unnecessary antibiotic use. By preventing influenza infections, which often prompt unnecessary antibiotic prescriptions, influenza vaccination reduces overall antibiotic demand. This reduction in antibiotic use helps preserve their effectiveness against bacterial infections, potentially limiting antimicrobial resistance. Thus, promoting universal influenza immunization represents a valuable strategy for reducing antimicrobial resistance through decreased antibiotic consumption.

Discussion

Impact of Vaccination as a Strategy Against AMR

Vaccination has had a significant impact on AMR and infection prevention globally. Vaccines have proven to be an effective tool in preventing a wide variety of infectious diseases, which in turn reduces the need for prescribing antibiotics. This helps to lower the incidence of bacterial infections and, therefore, reduces the likelihood of bacteria developing resistance to antibiotics [19,24].

Vaccines such as DTaP (diphtheria, tetanus, and pertussis), pneumococcal, and influenza are particularly effective in reducing the prevalence of bacterial infections. Conjugate pneumococcal vaccines have been linked to a decrease in the incidence of infections caused by drug-resistant Streptococcus pneumoniae (DRSP) [25].

The incidence of IPD is significantly higher among individuals under two years old and those over 65 years old. For instance, the incidence rate among people aged 75-84 years is 29.0 cases per 100,000, with a mortality rate of 4.53 per 100,000. Vaccination has notably reduced IPD rates. After the introduction of the PCV7 vaccine in 2000, the rates of disease from the seven serotypes covered by this vaccine in children under five years old drastically dropped from around 80 cases per 100,000 people to less than one case per 100,000 by 2007 [25]. Similar evidence was found in a study done in South Africa [12]. Furthermore, the introduction of PCVs decreased penicillin-resistant infections by 82% and ceftriaxone-resistant infections by 85% [20].

By lowering the incidence of infectious diseases, vaccines help minimize the use of antibiotics, which in turn, as mentioned above, reduces the risk of developing AMR. This is crucial since the excessive and inappropriate use of antibiotics is one of the main causes of AMR [19]. The introduction of conjugate pneumococcal vaccines has significantly reduced the rates of antibiotic-resistant invasive diseases. Between 1998-1999 and 2008, the rates of penicillin-non-susceptible invasive pneumococcal infections decreased by 64% in children under five years old and by 45% in adults over 65 years old [25].

Vaccination is most beneficial for the more vulnerable groups, such as children, the elderly, and those with compromised immune systems, thereby reducing the risk of serious infections that might require antibiotic treatment [26].

In the specific case of Costa Rica, the impact of vaccination on AMR and infection prevention has been significant. The country has robust vaccination programs and widespread access to vaccines, which have helped to reduce the incidence of vaccine-preventable diseases and, therefore, the use of antibiotics. This, in turn, has positively impacted AMR by reducing bacteria's exposure to these drugs. However, it is important to continue promoting proper vaccination and responsible antibiotic use to maintain these benefits over the long term [3,27].

Vaccines play a crucial role in decreasing disease prevalence and reducing dependency on antibiotics, underscoring their significance in antimicrobial stewardship programs. Additionally, the creation of new vaccines and the execution of vaccination protocols indirectly boost ASP initiatives [10,12]. For instance, reduced antibiotic consumption observed after starting vaccination programs or increasing vaccine coverage, with decreases from 5% to 10% in randomized trials to over 50% in epidemiological studies, indicates that immunization programs can lower antibiotic use. Consequently, checking vaccination status should be a standard practice in patient care [11].

The implementation of universal flu vaccination and vaccinating mothers postnatally resulted in a reduction in antibiotic prescriptions. The authors cited this as definitive proof supporting the routine inclusion of annual influenza vaccinations [13]. Influenza vaccination resulted in a 64% decrease in antibiotic prescriptions for respiratory conditions, highlighting the success of vaccination efforts in reducing unnecessary antibiotic usage and combating AMR [23].

Vaccination Strategies by Antimicrobial Stewardship Programs

Vaccination strategies are designed to identify patients at higher risk of bacterial or viral infections and complications associated with AMR. Such groups include immunocompromised patients, the elderly, children, and those with chronic diseases. Vaccines against pneumococcus and influenza are typically administered to these populations, which are considered at greater risk because age and chronic illnesses increase the likelihood of acquiring diseases such as pneumonia. It is crucial to highlight that both influenza and pneumococcal vaccines should be administered to healthy and sick patients [26].

Vaccines play a crucial role in minimizing the use of antibiotics and reducing the development of resistant strains within the intestinal microbiota, emphasizing their fundamental importance in the ongoing battle against AMR [15]. ASPs are crucial for ensuring the proper use of antimicrobials and minimizing resistance. Incorporating vaccinations and other preventive strategies into regular healthcare practices is vital for enhancing patient outcomes and effectively combating AMR. This integration helps maintain the effectiveness of treatments and supports the overall health system's efforts to manage infections more sustainably [14].

An ASP intervention successfully vaccinated 172 inpatients against influenza by screening their records for eligibility, with further plans to offer vaccinations upon discharge. In addition, initiatives such as Antibiotic Awareness Week were organized to educate on and promote safe antibiotic practices. These efforts underscore the integration of vaccination strategies within ASPs, aiming to diminish antibiotic misuse and effectively combat AMR [16].

The WHO's Immunisation Agenda for 2030 underscores the critical need to deepen our understanding of how vaccines influence AMR and emphasizes the importance of further researching their role within ASPs. It highlights that both individual and combined vaccines have demonstrated synergistic effects in lowering the utilization of antimicrobials and effectively combating resistance [21]. Vaccination plays a crucial role in ASPs, effectively reducing antibiotic use and combating AMR. For example, the introduction of the pneumococcal conjugate vaccine PCV7 in the United States led to a significant 41.9% decrease in antibiotic prescriptions for otitis media. Subsequently, PCV13 is projected to prevent 16.3 million cases of this condition. Another study that was conducted in Canada, with the introduction of universal influenza vaccination in Ontario in 2000, resulted in a 64% reduction in antibiotic prescriptions for respiratory illnesses compared to other provinces that did not have universal vaccination. This reduction highlights the impact of influenza vaccination in minimizing unnecessary antibiotic use. The effectiveness of influenza vaccines in reducing antibiotic prescriptions is twofold: first, by preventing secondary bacterial infections that often complicate influenza, and second, by reducing the inappropriate prescription of antibiotics for viral infections [6].

Additionally, studies conducted globally following Hib conjugate vaccines where demonstrated substantial declines in β-lactamase-positive Hib strains. In Canada, where Hib vaccines were introduced in 1988, Hib disease rates plummeted from 2.6 cases per 100,000 (1986-1987) to 0.08 cases per 100,000 (2011-2015), illustrating the vaccine's impact [6].

ASPs are crucial in managing respiratory conditions. Particularly noteworthy is the role of vaccination against Haemophilus influenzae for patients suffering from chronic obstructive pulmonary disease (COPD). Studies have shown that vaccinating these patients can lead to up to an 80% reduction in antibiotic usage [22]. Expanding the coverage of influenza 8 vaccination could be key to reducing AMR at a national level [18].

Educating medical staff, nursing staff, and patients about the importance of vaccination as an integral part of the strategy to combat AMR is vital. This includes promoting annual vaccines, specific vaccines according to patient needs, and encouraging adherence to recommended vaccination schedules [21,28].

Additionally, it becomes important to review a patient's vaccination history upon admission to a hospital or health area. This is to ensure that, if there are any vaccines due according to current vaccination guidelines, they can be administered and properly recorded. It is imperative that patients ensure their immunizations are up to date. This includes vaccines against influenza, pneumococcus, and hepatitis B, among others [29].

ASPs can develop specific protocols for vaccinating hospitalized patients, establishing eligibility criteria, scheduling vaccinations during hospitalization, and following up after hospital discharge to ensure the completion of vaccination schedules [24,29]. Coordination with hospital infection control programs can ensure a comprehensive strategy that addresses both infection prevention and the optimization of antimicrobial use [30]. It is important for ASPs to regularly monitor and evaluate the effectiveness of vaccination strategies in hospitalized patients, including vaccine coverage, the reduction of preventable infections, and the decrease in the use of antimicrobials associated with these measures [30,31].

Vaccination Barriers

In Table 2, the most significant points for overcoming vaccination barriers are highlighted. These include the importance of implementing a strategy that educates and changes attitudes through awareness campaigns emphasizing the benefits and safety of vaccines. Additionally, it is crucial to improve logistics and administration by investing in adequate infrastructure for vaccine storage, optimizing distribution systems, and training personnel to efficiently handle vaccines and maintain updated records. Ensuring the availability of vaccines through precise inventory management, establishing agreements with multiple suppliers, and planning vaccination needs in advance are also essential steps. This implementation requires effective coordination between governments, health providers, and international organizations, along with a sustained financial commitment to guarantee long-lasting and effective improvements [9].

**Table 2:** Barriers to vaccination.

Knowledge and Attitudes	Logistical and Administrative Barriers	Vaccine Shortages
A lack of knowledge about vaccination recommendations and benefits, along with negative beliefs and attitudes towards vaccines, are significant barriers to patient vaccination [9].	There are logistical and administrative challenges in accessing vaccines, including issues with distribution, storage, and proper administration of vaccines in hospitals [9].	The scarcity or shortage of some vaccines can limit the ability to implement effective vaccination programmes in hospital settings [9].

Limitations

The studies included in this review primarily focused on short-term outcomes following vaccination interventions. This may restrict the understanding of the long-term effectiveness of vaccination strategies in combating AMR, particularly in terms of sustained decreases in antibiotic resistance. Predominantly, the articles reflect experiences from high-resource settings, potentially limiting the generalisability of findings to low- and middle-income countries where AMR patterns and healthcare infrastructure differ significantly. There is a notable lack of standardized vaccination protocols across hospitals, which may influence the efficacy of ASPs and the role of vaccinations in reducing AMR. This variability can affect the consistency of results and the implementation of effective vaccination strategies universally. The implementation and monitoring of vaccination strategies require substantial resources, and many healthcare settings, especially in lower-income regions, may face challenges in deploying these strategies effectively due to financial and logistical constraints.

## Conclusions

Vaccination programs globally indirectly contribute to mitigating AMR by reducing preventable diseases and antibiotic use. They also protect vulnerable groups, improving their health outcomes. Hospital vaccination strategies, coordinated by ASP, could be effective in healthcare services. However, the specific impacts and effectiveness of these strategies need further investigation and quantification in line with the study’s objectives and results.
